# Retraction: MicroRNA-9 inhibits high glucose-induced proliferation, differentiation and collagen accumulation of cardiac fibroblasts by down-regulation of TGFBR2

**DOI:** 10.1042/BSR-2016-0346_RET

**Published:** 2023-04-06

**Keywords:** cardiac fibrosis, high glucose, human cardiac fibroblasts, miRNA-9, TGFBR2

This Retraction follows an Expression of Concern relating to this article previously published by Portland Press.

This article is being retracted from *Bioscience Reports* at the request of the Editor-in-Chief and the Editorial Board following receipt of a notification from a reader, alerting the Editorial Board to various images that seem to appear in other papers by unrelated authors.

The Figure 6A a-tubulin blots share similarities with the Figure 2F a-tubulin blots from Chen et al. 2019 (doi: 10.3727/096504018X15251282086836), the Figure 5A TGFBR2 blots share similarities with the Figure 5A PIK3R3 bands from Sun et al. 2020 (doi: 10.1042/BSR20193867) and the Figure 2 blots share similarities with those from Figure 3B of Wang et al. 2018 (doi: 10.1186/s11658-018-0089-x). The authors have been contacted with regards to the retraction and have not responded to the Journal's queries or the concerns raised. Given the extent of the issues raised, the Editorial Board stand by the decision to retract the article.

